# Examining the Impact of COVID-19 on People With Dementia From the Perspective of Family and Friends: Thematic Analysis of Tweets

**DOI:** 10.2196/38363

**Published:** 2022-06-27

**Authors:** Juanita-Dawne R Bacsu, Megan E O'Connell, Allison Cammer, Soheila Ahmadi, Corinne Berger, Mehrnoosh Azizi, Rory Gowda-Sookochoff, Karl S Grewal, Shoshana Green, Sheida Knight, Raymond J Spiteri

**Affiliations:** 1 Department of Psychology University of Saskatchewan Saskatoon, SK Canada; 2 College of Pharmacy and Nutrition University of Saskatchewan Saskatoon, SK Canada; 3 Department of Computer Science University of Saskatchewan Saskatoon, SK Canada

**Keywords:** coronavirus 2019, COVID-19, Twitter, social media, dementia, Alzheimer disease, thematic analysis, aging, older adult, elderly population, caregiver, support service, peer support, online health community

## Abstract

**Background:**

The COVID-19 pandemic is taking a serious toll on people with dementia. Given the rapidly evolving COVID-19 context, policymakers and practitioners require timely, evidence-informed research to address the changing needs and challenges of people with dementia and their family care partners.

**Objective:**

Using Twitter data, the objective of this study was to examine the COVID-19 impact on people with dementia from the perspective of their family members and friends.

**Methods:**

Using the Twint application in Python, we collected 6243 relevant tweets over a 15-month time frame. The tweets were divided among 11 coders and analyzed using a 6-step thematic analysis process.

**Results:**

Based on our analysis, 3 main themes were identified: (1) frustration and structural inequities (eg, denied dignity and inadequate supports), (2) despair due to loss (eg, isolation, decline, and death), and (3) resiliency, survival, and hope for the future.

**Conclusions:**

As the COVID-19 pandemic persists and new variants emerge, people with dementia and their family care partners are facing complex challenges that require timely interventions. More specifically, tackling COVID-19 challenges requires revisiting pandemic policies and protocols to ensure equitable access to health and support services, recognizing the essential role of family care partners, and providing financial assistance and resources to help support people with dementia in the pandemic. Revaluating COVID-19 policies is critical to mitigating the pandemic’s impact on people with dementia and their family care partners.

## Introduction

The COVID-19 pandemic is taking a serious toll on people living with dementia. Beyond having 1 of the highest COVID-19 mortality rates [1], people with dementia have experienced challenges to their mental, physical, and cognitive health [2]. In attempts to mitigate the spread of the COVID-19 virus, governments have implemented various infection control measures, such as public curfews, social distancing protocols, regional lockdowns, required masking, quarantines, visitation bans, and travel restrictions [3].

Studies show that people with dementia and their family care partners (eg, a spouse, common-law partner, or other family member who provides care to a person with dementia) [4] have experienced major barriers to accessing health services and community-based supports during the first wave of the pandemic. More specifically, challenges to service use included reduced or terminated adult day programs, respite services, cognitive rehabilitation therapy, friendly visitor services, senior center programs, and limited knowledge of or access to technology [5-7]. In addition, people with dementia and their family care partners experienced a range of unintended consequences from COVID-19 lockdowns and infection control measures, such as social isolation, loss of informal supports, and care partner fatigue and burnout [8-11].

As the pandemic continues to unfold, new coronavirus variants, such as Omicron, continue to create challenges for health and support services [12], and future variants are expected [13]. With this rapidly changing situation, policymakers and practitioners require timely and comprehensive data to address the changing needs of at-risk population groups, including people living with dementia. More specifically, research-based evidence is needed to inform COVID-19 policies to help mitigate the impact of the pandemic on people with dementia and their family care partners. However, new publications on dementia continue to report findings primarily based on the early stages of the pandemic with little knowledge of the COVID-19 barriers over time [5,9,14,15].

Given the rapidly evolving COVID-19 context, conducting in-person research remains a critical challenge with frequent changes to public health orders ranging from regional lockdowns to travel bans [16]. With a daily average of 500 million tweets, Twitter provides a novel and timely means for people to share their COVID-19 experiences [17]. Specifically, health care providers, policymakers, family members, and friends of people with dementia have been using Twitter to share their opinions, experiences, and concerns about the pandemic [18,19]. Accordingly, the purpose of this study was to use Twitter to examine the COVID-19 impact on people with dementia from the perspective of their family members and friends.

## Methods

### Tweet Extraction

Tweets were collected using Twint, an advanced scraping tool that enables users to scrape tweets without the use of Twitter’s application programming interface. This feature enables Twint scrapers to avoid certain restrictions, such as the number of tweets scraped, the frequency and period of scrapes, and the requirement of a Twitter account [20]. Thus, Twint was used to asynchronously scrape tweets for the period of September 8, 2020, to December 8, 2021.

**Figure 1:**
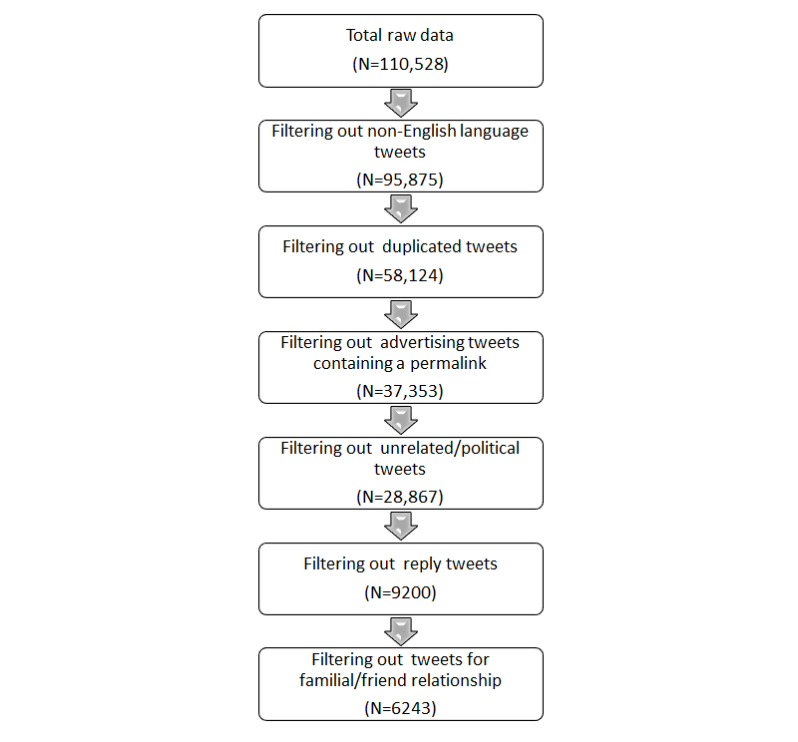
Twitter filters.

Given that existing Twitter studies already explore the early stages of the pandemic (ie, February 15-September 7, 2020, and March 17-24, 2020) on people with dementia [19,21], our study’s time frame focused further into the pandemic (ie, September 8, 2020, to December 8, 2021). Our full set of search terms included “dementia” OR “Alzheimer” used in combination with “COVID-19” OR “COVID” OR “Corona,” resulting in 110,528 tweets. We searched for tweets worldwide rather than focusing on tweets only from a certain country. Filters were applied to exclude non-English language tweets, reply tweets, advertising or spam tweets, and unrelated tweets, such as political tweets about “Trump” or “Biden” (refer to Figure 1). Only original tweets were cited, and replies to tweets were removed as they often were missing information and only contained half of the conversation. These filters are consistent with previous research analyzing Twitter data [18,22]. To improve the likelihood of scraping tweets that described personal COVID-19 experiences of dementia, we excluded tweets that did not include synonyms for familial relationships (eg, “mother,” “father,” “grandparent”) or friends or acquaintances (eg, “neighbor” and “pal”). The remaining 6243 tweets were put into a Microsoft Excel spreadsheet for data analysis.

### Data Analysis

Tweets were analyzed using a 6-step thematic analysis process [23]. First, 2 researchers (authors JB and MEO) read and reread 200 tweets to support familiarization with the data. Memos were used to document any new codes or ideas about the data to help supplement and revise an a priori code book [19]. This coding scheme comes from our previous Twitter research on the COVID-19 impact on dementia that was conducted at the early stages of the pandemic (ie, February 15-September 7, 2020) [19]. Second, the 2 researchers refined an existing coding scheme by deleting 2 codes (eg, questioning the cause of death and positive stories of survival) that were not identified within the current data. The final version of the revised codebook consisted of 9 codes: (1) death, (2) fear for the person with dementia’s health/well-being, (3) challenges and unmet needs, (4) separated/restricted visiting, (5) formal care provider/workforce issues, (6) supports described, (7) the informal care partner’s health/well-being, (8) stigma against dementia, and (9) irrelevant tweets (eg, no intersection of COVID-19 and dementia). A tweet code was included for irrelevant tweets that were not excluded by our filters and still required hand-coding to identify the tweets as irrelevant (eg, no intersection of COVID-19 and dementia). Approximately, 1200 tweets were coded as irrelevant. Third, the codebook was pilot-tested by having the full research team (ie, 11 coders) independently code the same set of 100 tweets, and then a meeting was held to discuss any coding questions or concerns. Fourth, a group coding exercise was organized, with the team collaboratively coding an additional set of the same 25 tweets to address issues of uncertainty. A final coding exercise was held by having each team member independently code another matching set of 25 tweets and then comparing codes to further support intercoder reliability. Fifth, after the practice coding exercises were completed, the 6243 tweets were divided among the 11 coders, with each coder receiving a different set of 567 (9.1%) tweets to code. The lead author oversaw and managed intercoder reliability by randomly reviewing 25% of all the coding, resulting in an intercoder reliability average of 83.4%. Any coding uncertainties or discrepancies were resolved through team meetings using group consensus. Sixth, once the coding was completed, 2 team meetings were held to discuss the patterns and relationships (eg, similarities, differences, overlap) among the codes, and theme piles were used to identify the overarching themes. The theme titles were reviewed and refined through group discussion to ensure clarity of the theme titles and properties.

### Rigor

We used 4 measures to support rigor in our study. First, a comprehensive audit trail was kept to document notes about the context of the research, methodological decisions (eg, code book revisions), and the 6-step thematic analysis process [24]. Second, each researcher used reflexive memoing to document interesting findings, similarities, differences, emerging patterns, and relationships throughout the coding process [23]. Third, our research team consisted of multidisciplinary researchers (eg, psychology, community health and epidemiology, computer science) with diverse skills and theoretical perspectives that provided a more insightful and nuanced approach to interpreting our study’s findings. Fourth, the first author managed intercoder reliability by randomly reviewing 25% of the codes to cross-check the data analysis, resulting in an intercoder reliability average of 83.4%.

### Ethical Considerations

Our research used publicly available tweets posted on Twitter. No interaction was made with the users who posted the tweets. We removed the Twitter handles and usernames to help protect the anonymity of the tweeters. Consequently, our Twitter research did not require review by an institutional ethics review board.

## Results

Drawing on our thematic analysis, 3 main themes were identified: (1) frustration and structural inequities (eg, denied dignity and inadequate supports), (2) despair due to loss (eg, isolation, decline, and death), and (3) resiliency, survival, and hope for the future.

### Frustration and Structural Inequities: Denied Dignity and Inadequate Supports

A predominant theme focused on frustration and structural inequities experienced by people with dementia and their care partners amidst the infection control policies to mitigate COVID-19. Family care partners reported that due to structural inequities, people with dementia were often revoked of their fundamental dignities and denied their basic rights. Family members spoke of restraint use, overmedication, dehydration, weight loss, neglect, elder abuse, ageism, isolation, no physician visits, vaccination challenges (delays or no access to COVID-19 vaccines), and understaffing in care facilities. The following tweets illustrate some of these issues:

…My Dad died Alone in Hospital with Alzheimers and they wouldnt let me see or talk to him. He was Overmedicated and Restrained. Thats Elder Abuse!

Is that in protocol of a hospital for an alzheimer patient to keep them tied as animals?? proper hygiene of a COVID patint was also neglected his dead body was in same t-shirt after 10 days of his admission to hospital…

The remote GP is also a fact in care homes, with at least one care home reporting that the local GP will not visit the dementia floor “because of Covid” unless it is an end of life situation…

Her father had dementia. She says he was tied up in his bed because there weren't enough staff even before covid.

Tweets described that COVID-19 policies failed to address the needs and showed a lack of regard for people with dementia. Family care partners discussed circumstances where restrictive policies went beyond necessary health precautions and compromised the health of people with dementia. For example, visitation bans and restriction of family presence within care facilities created barriers for people with dementia, especially for people who were unable to advocate for their personal health needs. Family members expressed frustration with instances where they were not allowed to accompany their loved one with dementia within ambulance transport, the hospital, or during treatment within acute care settings. This frustration with COVID-19 restrictions is demonstrated in the following tweets:

My Grandma had a fall this morning (she's okay just shaken up), she has dementia and had to be taken in the ambulance ON HER OWN because of covid. My aunty couldn't go with her. Yet we are allowed to go out and exercise with a group of 10 people. This is a load of crap.

So. the fella with dementia can't have his wife who has been isolating at home and double vaxxed accompany him for emerg appt on wound care but COVID partner can be there for birth. Is that not ageism at it's finest…

Many tweets identified financial challenges and inadequate supports during the pandemic. For example, some family care partners were unable to be employed while providing care, and others described financial issues, such as debt, unemployment, and costly medical insurance. Limited housing options for people with dementia were discussed within the community, and long-term care homes were described as being overwhelmed by COVID-19. These challenges are captured in the following tweets:

Being unemployed really sucks. I left my kids and jobs behind to come back to the UK to look after my father as his dementia has made life hard. Now it looks like we have more COVID restrictions coming in this week as well. Stay positive people and follow the damn rules!!!!

…I am the sole caregiver for my mom who has dementia. She has gotten pretty bad. It has been really difficult. I have no help and there are not a lot of services right now with Covid.

My step Mom died of covid in the nursing home yesterday. She had Alzheimer's. The nursing home is overwhelmed with covid 19. Sending in national guard because staff ill too. Not seeing this on the news…

### Despair Due to Loss: Isolation, Decline, and Death

Psychological loss was at the core of many tweets. Many people lamented the impacts of social distancing on the lives of people with dementia. Isolation, a consequence of pandemic mitigation strategies, was blamed for accelerating cognitive, psychological, and physical decline.

Just FaceTimed my gma for the first time in a while. Been in covid lockdown at ret. home for 8mos. Her Alzheimers has accelerated. Cant see neighbors. No visitors. Just TV in her room. They allowed her to finally leave cus she wouldnt stop crying. Doesnt know anybody anymore.

Isolation from pandemic mitigation strategies were also blamed for hastening death.

COVID killed my mother Not inthe hospital gasping for air, this 90 year old nursing home resident with mild dementia was moved out of her unit and placed on a wing where she knew no one then shut shut off visitors for safety 6 months later she gave up, mom died from loneliness

Some simply described the experiences of losing a loved one with dementia due to COVID-19.

Today is a sad day for cabbit, my father is no longer with us. Alzheimer's took his mind, but covid took his body. I'll miss you daddy.

Many tweets, however, conveyed complicated reactions to their loss of a loved one who was living with dementia to COVID-19. These reactions, which span anger, helplessness, and a sense of futility, seem to be factors that could interrupt the process of making meaning from loss and thereby disrupt the grieving process and create psychological vulnerability to future mental health challenges (eg, from a depressed mood).

Hes had dementia, a few strokes, and heart surgery, but what finally did him in was a combo of COVID and the selfishness of some I used to call family. This week felt like a long panic attack but with anger. We knew wed lose him, but him being taken feels so much worse.

Some lamented the challenges of concomitant dementia and active COVID-19 infection requiring hospitalization or medical interventions that made the loss even more challenging to process, accept, and make meaning from.

My father died this morning. He had a lot of medical issues, but COVID sent him to the hospital, which meant we werent able to see him during his last weeks. Considering he had Alzheimers, I cant imagine how terrifying that mustve been. Wear the damn masks and stay inside.

My grandma had dementia, which is always terminal. Covid-19 took her last good years away and she died in a coma on a ventilator and no one could touch her. No one dies a good, peaceful death if they have covid-19.

Many expressed anger when describing their experiences with losing a loved one with dementia due to COVID-19. Although anger was reported earlier in the pandemic related to mask usage and lack of social distancing [19], as the medical science has evolved to illuminate ways to prevent or mitigate COVID-19 infection, the nature of the tweeters’ anger has similarly evolved. For some, the anger was directed at those who were unvaccinated, those who interacted without wearing masks, and government-led mitigation strategies. These tweets convey a sense of helplessness and struggles to find meaning from deaths that these tweeters felt could have been avoided.

This covid shit is no joke at all. My mom mom passed this morning. After a total of 20+ years living with a heart AND kidney transplant + recently being diagnosed w/ dementia, my gmom fought long and hard just to be taken out by something that could have been prevented…

The experiences of losing someone with dementia who resided in long-term care were described. Many of these conveyed the sense of helplessness and sense of tragedy at the numerous cases of COVID-19 in long-term care, further compounding the sense of loss and likely to interfere with the process of grieving.

My paternal grandfather passed away in October, 1 month after being admitted to a nursing home rampant with COVID b/c Drs decided my 88 year old grandmother could not longer care for him and his worsening dementia at home. It was the only facility their insurance would cover...

I left mum 13/3 in her dementia care home - she died 1/4 one of many who died after hospital discharged covid positive patients into homes. The voice of the voiceless #neverforgotten

Disrupted grieving rituals were also commonly described. Adherence to grieving rituals helps with reminiscence through a shared experience with others [25]. Sharing the loss with others creates a sense of universality in the experience and is also psychologically healing [26]. The pandemic has disrupted the shared grieving process of gathering at funerals, potentially making the grieving process unduly prolonged and creating psychological vulnerability to future mental health concerns.

I literally just had to attend my Grandfathers funeral via zoom. Hes been in stable condition over the last few years (Alzheimers and Dementia) but his nursing home had an outbreak of COVID and he passed a few days later. How is this fair? Were never prepared for death.

Today, the final goodbye to Grandad . 96 years old. Alzheimers robbed him of 10 years ,COVID ravaged him in 3 days. 30 in church only 15 at the crematorium…not allowed to see him in chapel of rest, no sharing our grief with family, no remembering him at a wake. Sad and angry

1 year ago my mom died of Alzheimers. Because of Covid she never had a funeral. I feel like Ive failed her

### Resiliency, Survival, and Hope for the Future

Another predominant theme was resiliency, survival, and hope for the future. Resiliency was often discussed in terms of one’s ability to fight, survive, and recover from the COVID-19 virus. Some tweets of people with dementia and their family members described psychological resilience, mental toughness, and determination to fight against COVID-19. More specifically, these tweets discussed the pandemic in terms of going to a battle or being in a fight. Many tweets also described resiliency in terms of surviving COVID-19 against multiple odds. For example, these tweets highlighted that despite facing numerous challenges, they still fought the virus and won. Resiliency against the virus is highlighted in tweets such as:

My nana had the vaccine yesterday shes 86, has dementia, had both her hips replaced, had a stroke and a cornea transplant AND fought covid in an english hospital when she only speaks portuguese and came out stronger on the other side, i am so thankful for her.

…My mom has suffered so many indignities with Alzheimers. Shes fought through them all. 17 cases of Covid in her unit and she didnt get it. My mom is determined to leave this earth on her terms. Not Covids.

People also shared tweets on factors that they perceived as being linked to COVID-19 resiliency and recovery against the virus. Specifically, these factors included different aspects, such as self-care (eg, eating well and staying positive), formal supports (eg, health care), lifestyle factors (eg, physical activity and sleep/rest), and getting the COVID-19 vaccine. The following tweets illustrate factors that people credited with recovery from the virus:

My mother 77 with Alzheimer because of her veganist diet survived corona. Me too. By just eating and drinking more. To be optimist. Fruits are the secret. Normal sleep. A bit sleep during the day 2 hours during corona. 5 days cure. Keep walking. Lots of sunshine please. Open windows.

My mother who is 88 and lives with severe dementia caught COVID in August came through some days when we were told the end was nigh and is back home getting stronger and stronger. This is down to the vaccine and great care at Queens Hospital Romford @NHSuk

Many tweets discussed a strong sense of relief and hope for the future. These tweets were often strongly linked to the COVID-19 vaccine. For example, people expressed relief and elation after receiving their COVID-19 vaccination. Many conveyed that the vaccines helped them overcome anxiety and fear that they or their loved ones with dementia would not be protected against the virus. Family members and care partners described breathing a sigh of relief after their family members with dementia were vaccinated, especially in care facilities. Relief is captured in the following tweets:

We just received word that the Alzheimers memory care facility my dad lives in is getting vaccines. I cannot express what a relief this is for my mom. They were Covid free until just before Thanksgiving. Weve been holding our breath for 3 weeks, now we can exhale.

My grandmother gets her vaccine on the 28th. She has severe dementia and lives in a memory care facility thats had several COVID diagnoses, so Im beyond relieved…Im glad to know shell be protected.

My mom (end stage cancer) gets her Pfizer vaccine today. My dad (Alzheimers) got his a few weeks ago. I want to celebrate…Ive had a year of low level panic for them. My heart is with all those who lost their loved ones to Covid. #vaccine

Dad - who turns 75 in a week-and-a-half, has advanced Parkinsons (and dementia) and lives in aged care - just got his first #COVID19Vaccine today. Relief is pretty much the only thing Im feeling about it. #Covid

People described hope by sharing and envisioning their future plans related to the easing of COVID-19 restrictions. For example, many looked forward to reconnecting in person with people with dementia in care facilities. These tweets often described long-awaited anticipation to engage in face-to-face visits, partake in activities, and provide hugs and a human touch. This sense of hope and anticipation to reconnect is highlighted in tweets such as:

The news that I can go see my Gran next week makes me so so happy. So much lost time over the past year with COVID and over that time her dementia has worsened and now wont remember me but to be able to give her a huge hug means more than anything. Family first always

Covid restrictions lifted at girlfriends moms alzheimer dementia care facility. Busting her out today for a pre mother's day ride on Verde Valley Railroad…

I talk to my mom almost daily and before my dads Alzheimers got bad, the same. As soon as Covid restrictions lift, Ill be back visiting him weekly again.

## Discussion

### Principal Findings

Our study aimed to examine the COVID-19 impact on people with dementia from the perspective of their family members and friends. Understanding COVID-19 challenges is critical to informing targeted policies, programs, and resources to support people living with dementia. Drawing on thematic analysis, our study identified 3 main themes: (1) frustration and structural inequities (eg, denied dignity and inadequate supports), (2) despair due to loss (eg, isolation, decline, and death), and (3) resiliency, survival, and hope for the future. Our findings have important COVID-19 policy and research implications.

Based on our findings, we believe that policymakers must eliminate structural inequities and barriers to health care for people with dementia due to the pandemic. More specifically, governments must revisit COVID-19 policies and infection control measures (eg, visitation bans, restricting family presence, confinement, quarantine, and lockdowns) to ensure that people with dementia and their care partners have equitable access to health care and support services. Too often, tweets highlighted issues of restraint use, overmedication, dehydration, weight loss, neglect, ageism, isolation, psychological deterioration, cognitive decline, no physician visits, vaccination challenges (delays or no access to COVID-19 vaccines), and understaffing in care facilities, especially in long-term care homes. Banerjee and Estabrooks [27] assert that long-term care residents, including people with dementia and staff, were often last in line for COVID-19 testing, last to receive personal protective equipment, and directed not to send their residents to hospitals in order to keep the hospital beds open for others. Similarly, the existing literature shows that compared to prepandemic years, people with dementia in long-term care homes have experienced increased use of antipsychotic medications [28], increased confinement and social isolation [29-31], reduced physician visits, decreased access to specialist services, and reduced hospital transfers for illness [32]. Consequently, improving COVID-19 outcomes for people with dementia requires addressing structural inequities in health and support services.

Policymakers need to recognize the essential role of family care partners for people with dementia during the pandemic. Many tweets highlighted care partner challenges related to health care navigation, barriers in health care communication, and COVID-19 visitation restrictions that inhibited their support for people with dementia in care facilities (eg, hospitals, emergency departments, medical clinics, and long-term care homes). Moreover, visitation restrictions were often blamed for psychological loss and accelerating cognitive, psychological, and physical decline in people with dementia. Consequently, COVID-19 policies must be loosened to allow family care partners to accompany, access, and provide necessary support to people with dementia in care facilities. In the United States alone, over 16 million people provide unpaid care to people with dementia at an economic value of US $244 billion [33]. Furthermore, numerous tweets identified financial challenges, issues of economic uncertainty, and inadequate resources among people with dementia and their family care partners. These issues of financial hardship among people with dementia and their care partners are reflected in the existing COVID-19 literature [34,35]. Accordingly, policies and programs are needed to provide financial assistance and resources to help support and sustain people with dementia and their family care partners.

Although many tweets highlighted COVID-19 challenges, some tweets also discussed resiliency and hope for the future. Numerous tweets described people with dementia beating the virus and surviving against multiple odds. People also shared a strong sense of optimism and hope related to COVID-19 vaccines and the easing of restrictions. Many of these tweets discussed future plans to engage in face-to-face visits, partake in activities, and provide hugs and a human touch. These findings suggest that resiliency and hope may play an important role for people with dementia and their care partners during times of adversity, such as a pandemic. However, further research is needed to examine this area.

### Limitations

Although a rigorous process was undertaken to perform this study, our research includes limitations. For example, our study did not collect any sociodemographic information about the tweeters. Accordingly, it is difficult to make specific inferences or generalizations about our findings in relation to age, ethnicity/culture, and gender or sex. Moreover, our study did not collect data based on the tweeter’s country or geographic location. Twitter research with sociodemographic and geographical components would provide more insight into COVID-19’s impact in relation to sociodemographic and geographical characteristics. However, statistics show that Twitter users are from different countries across the world, with a leading number of users (in the millions) in the United States (76.9), Japan (58.95), India (23.6), Brazil (19.05), Indonesia (18.45), and the United Kingdom (18.4) [36]. Consequently, it is interesting to note that our themes were consistent across our data.

Another limitation of our Twitter study is that it is based on cross-sectional research rather than longitudinal data. For example, longitudinal data over different years would provide greater insight into COVID-19 policy responses, changes, and implications over time. Accordingly, further research with longitudinal data would provide useful information on the temporal aspects of different policies and their related outcomes (eg, social, emotional, cognitive, and physical health).

In addition, our research is limited by our scraping strategy that focused on tweets with familial relationships, friends, and acquaintances. Although we aimed to have an inclusive scraping strategy, it is possible that we may have missed relevant data on the COVID-19 impact on people with dementia. For example, future research could use a broader search filter to ensure no relevant findings were overlooked. Moreover, further Twitter research is needed to focus on the lived experiences of people with dementia during the pandemic.

Lastly, each tweet is limited to Twitter’s 280-character limit. Consequently, it is possible that we might be missing important background details or contextual factors needed to fully understand the tweet’s meaning. To help mitigate this issue, we held research team meetings to discuss and clarify any coding uncertainties by using group consensus.

### Conclusion

Given the rapidly evolving COVID-19 context, policymakers and practitioners require timely, evidence-informed research to address the changing needs of marginalized population groups, including people with dementia. Using Twitter data, the objective of our study was to examine the COVID-19 impact on people with dementia from the perspective of their family members and friends. Our study identified 3 main themes: frustration and structural inequities (eg, denied dignity and inadequate supports), despair due to loss (eg, isolation, decline, and death), and resiliency, survival, and hope for the future.

As the pandemic persists and new variants emerge, people with dementia and their family care partners are facing complex challenges that require timely interventions. However, addressing these challenges is neither a straightforward nor a simple task. Rather, tackling these challenges requires interventions informed by lived experiences and evidence-based research. More specifically, tackling COVID-19 challenges requires revisiting infection control policies to ensure equitable access to health and support services, recognizing the essential role of family care partners, and providing financial assistance and resources to help support people with dementia throughout the pandemic. Re-evaluating COVID-19 policies is critical to mitigating the pandemic’s impact on people with dementia and their family care partners.
